# Crystal structure of 1,6-di­thia­cyclo­deca-*cis*-3,*cis*-8-diene (DTCDD)

**DOI:** 10.1107/S1600536814023319

**Published:** 2014-10-31

**Authors:** Russell G. Baughman, Molly C. Delanty, Michael F. Ortwerth

**Affiliations:** a Department of Chemistry, Truman State University, Kirksville, MO 63501-4221, USA; b Office of Special Medical Programs, Food and Drug Administration, Silver Spring, MD 20993-0002, USA

**Keywords:** crystal structure, 1,6-di­thia­cyclo­deca-*cis*-3,*cis*-8-diene, DTCDD

## Abstract

The title compound, C_8_H_12_S_2_ (trivial name DTCDD), was obtained as a side product of the reaction between *cis*-1,4-di­chloro­but-2-ene and sodium sulfide. The asymmetric unit consists of one-quarter of the mol­ecule (S site symmetry 2) and the complete mol­ecule has 2/*m* (*C*_2*h*_) point symmetry with the C=C bond in an *E* conformation. The geometry of the title compound is compared to those of a chloro derivative and a mercury complex.

## Related literature   

The structure of the compound having the ethyl­inic H atoms replaced by Cl atoms has been reported (Eaton *et al.*, 2002 ▶) as has one where the title compound is ligated to Hg atoms (Cheung & Sim, 1965 ▶).
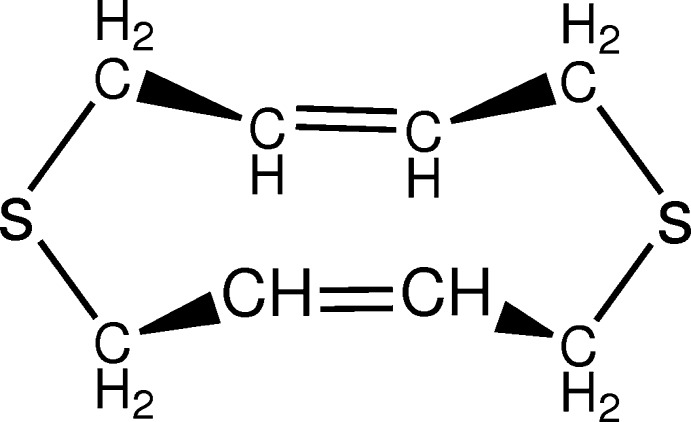


## Experimental   

### Crystal data  

C_8_H_12_S_2_
*M* *_r_* = 172.31
Orthorhombic, 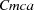

*a* = 13.5706 (6) Å
*b* = 7.5329 (4) Å
*c* = 8.4303 (4) Å
*V* = 861.80 (7) Å^3^
*Z* = 4
Mo *K*α radiation
μ = 0.54 mm^−1^
*T* = 293 K
0.43 × 0.40 × 0.17 mm

### Data collection  

Bruker P4 diffractometer
Absorption correction: integration (*XSHELL*; Bruker, 1999 ▶) *T*_min_ = 0.676, *T*_max_ = 0.845
707 measured reflections
509 independent reflections
398 reflections with *I* > 2σ(*I*)
*R*_int_ = 0.027
3 standard reflections every 100 reflections intensity decay: 1.0%

### Refinement  

*R*[*F* ^2^ > 2σ(*F* ^2^)] = 0.034
*wR*(*F* ^2^) = 0.090
*S* = 1.07
509 reflections
24 parameters
H-atom parameters constrained
Δρ_max_ = 0.19 e Å^−3^
Δρ_min_ = −0.22 e Å^−3^

### 

Data collection: *XSCANS* (Bruker, 1996 ▶); cell refinement: *XSCANS*; data reduction: *XSCANS*; program(s) used to solve structure: *SHELXS86* (Sheldrick, 2008 ▶); program(s) used to refine structure: *SHELXL97* (Sheldrick, 2008 ▶); molecular graphics: *SHELXTL/PC* (Sheldrick, 2008 ▶); software used to prepare material for publication: *SHELXTL/PC* and *SHELXL97*.

## Supplementary Material

Crystal structure: contains datablock(s) I, global. DOI: 10.1107/S1600536814023319/hb7285sup1.cif

Structure factors: contains datablock(s) I. DOI: 10.1107/S1600536814023319/hb7285Isup2.hkl

Supporting information file. DOI: 10.1107/S1600536814023319/hb7285Isup3.cml

**Fig. 1.**
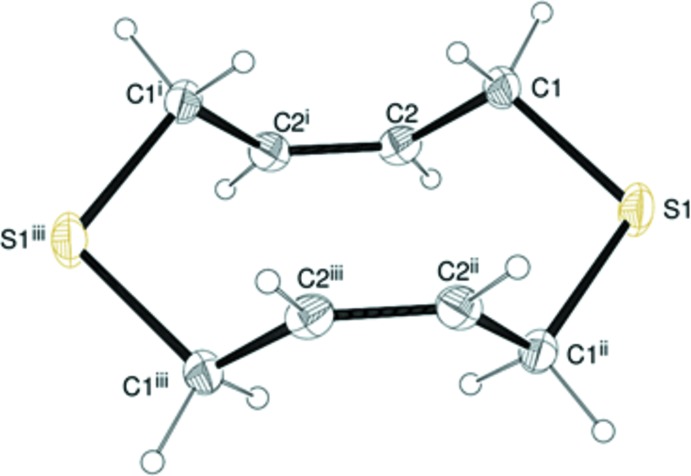
The molecular structure of DTCDD with displacement ellipsoids drawn at the 30% probability level. Symmetry codes: (i) 1-x, y, z; (ii) x, 1-y, 1-z; (iii) 1-x, 1-y, 1-z.

. DOI: 10.1107/S1600536814023319/hb7285fig1.tif
The mol­ecular structure of DTCDD with displacement ellipsoids drawn at the 30% probability level. Symmetry codes: (i) 1-x, y, z; (ii) x, 1-y, 1-z; (iii) 1-x, 1-y, 1-z.

**Fig. 2.**
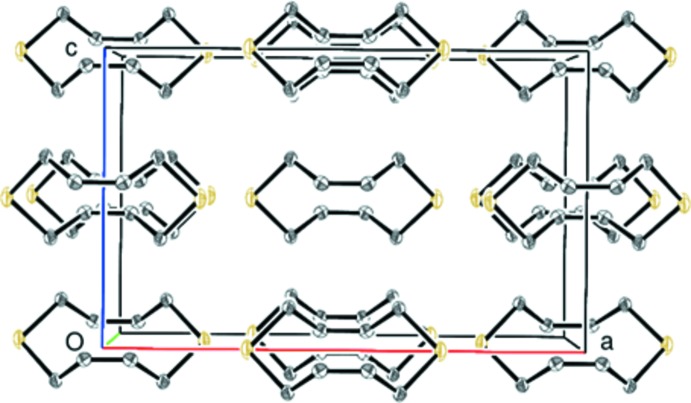
The unit-cell packing in DTCDD viewed down the b-axis.

. DOI: 10.1107/S1600536814023319/hb7285fig2.tif
The unit-cell packing in DTCDD viewed down the b-axis.

CCDC reference: 1030564

Additional supporting information:  crystallographic information; 3D view; checkCIF report

## Figures and Tables

**Table 1:** Comparison of selected geometric parameters (, ) for the title and two similar compounds All three compounds crystallize in centrosymmetric space groups, thus there are values for all torsion angles.

Atoms*^*a*^*	DTCDD	Cl derivative*^*b*^*	Hg ligated^*c*,^ ^*d*^
S1C1	1.8177(18)	1.809(2), 1.805(2)	1.87
C1C2	1.484(3)	1.494(3)	1.60
C2C2^i^	1.333(3)	1.326(3)	1.30
			
C1S1C1	101.52(11)	101.63(10)	103
C2C1S1	112.93(13)	115.28(15), 114.69(14)	110
C2^*f*^C2C1	127.18(9)	125.91(17), 125.64(19)	128
			
C2C1S1C1^ii^	59.88(11)	61.75, 64.51*^*d*^*	63.17, 54.95*^*d*^*
S1C1C2C2^i^	122.78(19)	119.82, 123.28*^*d*^*	121.22, 127.77*^*d*^*

Notes: (*a*) This work’s labeling; (*b*) Eaton *et al.* (2002 ▶); (*c*) Cheung Sim (1965 ▶); (*d*) from the CSD (Allen, 2002 ▶). Symmetry codes: (i) 1*x*, *y*, *z*; (ii) *x*, 1*y*, 1*z*.

## supplementary crystallographic information

### S1. Structural commentary

During a study of hydro­desulfurization, the reaction of *cis*-1,4-di­chloro-2-butene and sodium sulfide yielded 1,6-di­thia­cyclo­deca-*cis*-3,*cis*-8-diene ("DTCDD)") as a side product. Since its structure is not listed in the Cambridge Structural Database (Allen, 2002), although the Cl derivative (Eaton *et al.*, 2002) and Hg-ligated form (Cheung and Sim, 1965) are, it was decided to perform the single-crystal structural analysis of DTCDD. The asymmetric unit of DTCDD is C_2_H_3_S_0.5_, which then generates three more symmetry elements within the 22-atom molecule (C_8_H_12_S_2_) (Fig. 1) in the *Cmca* unit cell which contains four molecules (Fig. 2).

Comparisons of DTCDD with the Cl and Hg derivatives give some insight into the nature of the systems. The C═C bonds for all three compounds exhibit the *E* isomer (*cf.* Fig. 1). The Hg data were derived from film data, so precise comparisons of distances and angles is somewhat limited, although some conclusions may still be drawn. If the s.u.'s in Hg distances and angles are assumed to be ~0.02 Å and 1°, respectively, the three compounds have many similar distances and angles ≤ 3σ (Table 1). There are, however, a few noteworthy exceptions.

The S1—C1 bond lengths in DTCDD and the Cl derivative are within 3σ of each other while the C1—C2 distance in the Hg complex is ~6σ greater than the other two. The two C2—C1—S1 angles in DTCDD and the Cl derivative differ by as much as 16σ; the same angle in the Cl derivative may differ by as much as 5σ (5°) from the Hg derivative, while the DTCDD and Hg derivative angles are essentially the same (≤3σ). A difference of as much as 8σ is noted between the C2═C2—C1 angles in DTCDD and the Cl derivative, while the Hg analog angle is within 3σ of both of the other compounds. Many of these differences may likely be attributed to the presence of the Cl's on all four C2's only in the Cl derivative.

### S2. Synthesis and crystallization

DTCDD is a side product of the reaction of *cis*-1,4-di­chloro-2-butene and sodium sulfide in MeOH/DMSO. DTCDD was slowly recrystallized from a solution in pentane to yield colourless parallelepipeds.

### S3. Refinement

Crystal data, data collection and structure refinement details are summarized in Table 1. Approximate positions of the H atoms were first obtained from a difference map, then placed into "ideal" positions. Bond lengths were constrained at 0.93 Å (AFIX 43) for the ethyl­enic H and at 0.97 Å (AFIX 23) for the methyl­enic H's. *U*_iso_(H) were fixed at 1.2 *U*_eq_(parent).

In the final stages of refinement, 4 reflections with very small or negative *F_o_*'s were deemed to be in high disagreement with their *F_c_*'s and were eliminated from final refinement.

### Crystal data

**Table:**

C_8_H_12_S_2_	*F*(000) = 368
*M**_r_* = 172.31	*D*_x_ = 1.328 Mg m^−^^3^
Orthorhombic, *C**m**c**a*	Mo *K*α radiation, λ = 0.71073 Å
Hall symbol: -C 2bc 2	Cell parameters from 100 reflections
*a* = 13.5706 (6) Å	θ = 10.8–22.2°
*b* = 7.5329 (4) Å	µ = 0.54 mm^−^^1^
*c* = 8.4303 (4) Å	*T* = 293 K
*V* = 861.80 (7) Å^3^	Parallelepiped, colorless
*Z* = 4	0.43 × 0.40 × 0.17 mm

### Data collection

**Table:**

Bruker P4 diffractometer	398 reflections with *I* > 2σ(*I*)
Radiation source: normal-focus sealed tube	*R*_int_ = 0.027
Graphite monochromator	θ_max_ = 27.5°, θ_min_ = 3.0°
θ/2θ scans	*h* = −1→17
Absorption correction: integration (*XSHELL*; Bruker, 1999)	*k* = −1→9
*T*_min_ = 0.676, *T*_max_ = 0.845	*l* = −10→1
707 measured reflections	3 standard reflections every 100 reflections
509 independent reflections	intensity decay: 1.0%

### Refinement

**Table:**

Refinement on *F*^2^	Primary atom site location: structure-invariant direct methods
Least-squares matrix: full	Secondary atom site location: difference Fourier map
*R*[*F*^2^ > 2σ(*F*^2^)] = 0.034	Hydrogen site location: difference Fourier map
*wR*(*F*^2^) = 0.090	H-atom parameters constrained
*S* = 1.07	*w* = 1/[σ^2^(*F*_o_^2^) + (0.0314*P*)^2^ + 0.6276*P*] where *P* = (*F*_o_^2^ + 2*F*_c_^2^)/3
509 reflections	(Δ/σ)_max_ < 0.001
24 parameters	Δρ_max_ = 0.19 e Å^−^^3^
0 restraints	Δρ_min_ = −0.22 e Å^−^^3^

### Special details

**Table:**

Geometry. All s.u.'s (except the s.u. in the dihedral angle between two l.s. planes) are estimated using the full covariance matrix. The cell s.u.'s are taken into account individually in the estimation of s.u.'s in distances, angles and torsion angles; correlations between s.u.'s in cell parameters are only used when they are defined by crystal symmetry. An approximate (isotropic) treatment of cell s.u.'s is used for estimating s.u.'s involving l.s. planes.
Refinement. Refinement of *F*^2^ against ALL reflections. The weighted *R*-factor, *wR*, and goodness of fit, *S*, are based on *F*^2^, conventional *R*-factors, *R*, are based on *F*, with *F* set to zero for negative *F*^2^. The threshold expression of *F*^2^ > 2σ(*F*^2^) is used only for calculating *R*-factors(gt) *etc*. and is not relevant to the choice of reflections for refinement. *R*-factors based on *F*^2^ are statistically about twice as large as those based on *F*, and *R*-factors based on ALL data will be even larger.

### Fractional atomic coordinates and isotropic or equivalent isotropic displacement parameters (Å^2^)

**Table:**

	*x*	*y*	*z*	*U*_iso_*/*U*_eq_	
S1	0.30007 (4)	0.5000	0.5000	0.0595 (3)	
C1	0.38479 (12)	0.6100 (3)	0.3650 (2)	0.0431 (5)	
H1A	0.4246	0.5210	0.3119	0.052*	
H1B	0.3472	0.6724	0.2846	0.052*	
C2	0.45087 (12)	0.7380 (2)	0.4462 (2)	0.0400 (4)	
H2	0.4204	0.8281	0.5034	0.048*	

### Atomic displacement parameters (Å^2^)

**Table:**

	*U*^11^	*U*^22^	*U*^33^	*U*^12^	*U*^13^	*U*^23^
S1	0.0253 (3)	0.0766 (6)	0.0767 (6)	0.000	0.000	0.0329 (5)
C1	0.0316 (8)	0.0521 (11)	0.0455 (9)	0.0009 (8)	−0.0024 (7)	0.0113 (8)
C2	0.0460 (10)	0.0333 (8)	0.0406 (8)	0.0076 (8)	0.0049 (8)	0.0055 (7)

### Geometric parameters (Å, º)

**Table:**

S1—C1	1.8177 (18)	C1—H1B	0.9700
S1—C1^i^	1.8177 (18)	C2—C2^ii^	1.333 (3)
C1—C2	1.484 (3)	C2—H2	0.9300
C1—H1A	0.9700		
			
C1—S1—C1^i^	101.52 (11)	S1—C1—H1B	109.0
C2—C1—S1	112.93 (13)	H1A—C1—H1B	107.8
C2—C1—H1A	109.0	C2^ii^—C2—C1	127.18 (9)
S1—C1—H1A	109.0	C2^ii^—C2—H2	116.4
C2—C1—H1B	109.0	C1—C2—H2	116.4
			
C1^i^—S1—C1—C2	−59.88 (11)	S1—C1—C2—C2^ii^	122.76 (9)

Symmetry codes: (i) *x*, −*y*+1, −*z*+1; (ii) −*x*+1, *y*, *z*.
