# Supplementary figures and images for: Crystal structure of 1,6-di­thia­cyclo­deca-cis-3,cis-8-diene (DTCDD)
Source: Acta Crystallogr Sect E Struct Rep Online. 2014 Oct 31;70(Pt 11):o1218. doi: 10.1107/S1600536814023319 (PMC4257335; doi:10.1107/S1600536814023319)

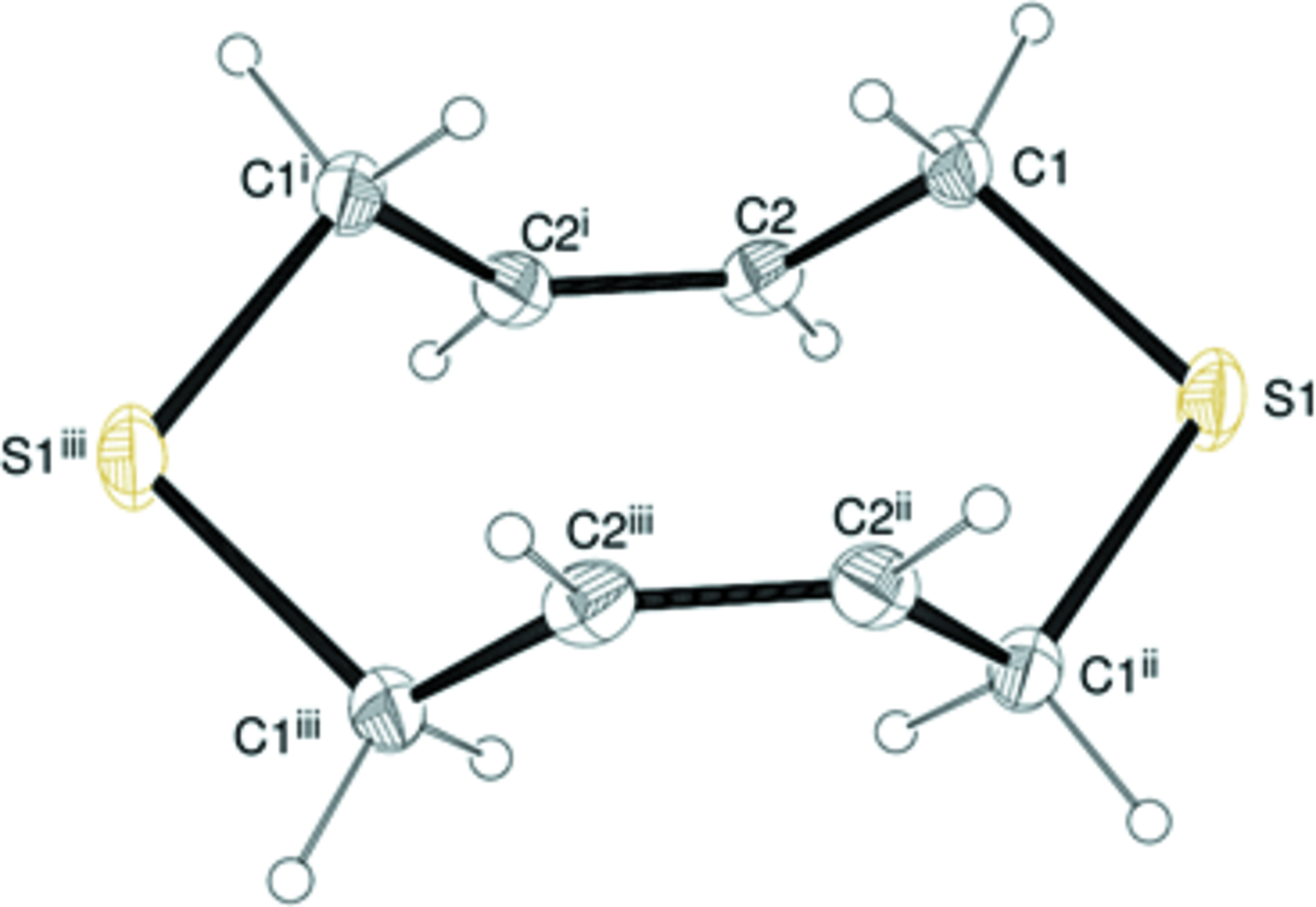

Supplement: Supplementary file 4 [file e-70-o1218-fig1.tif]

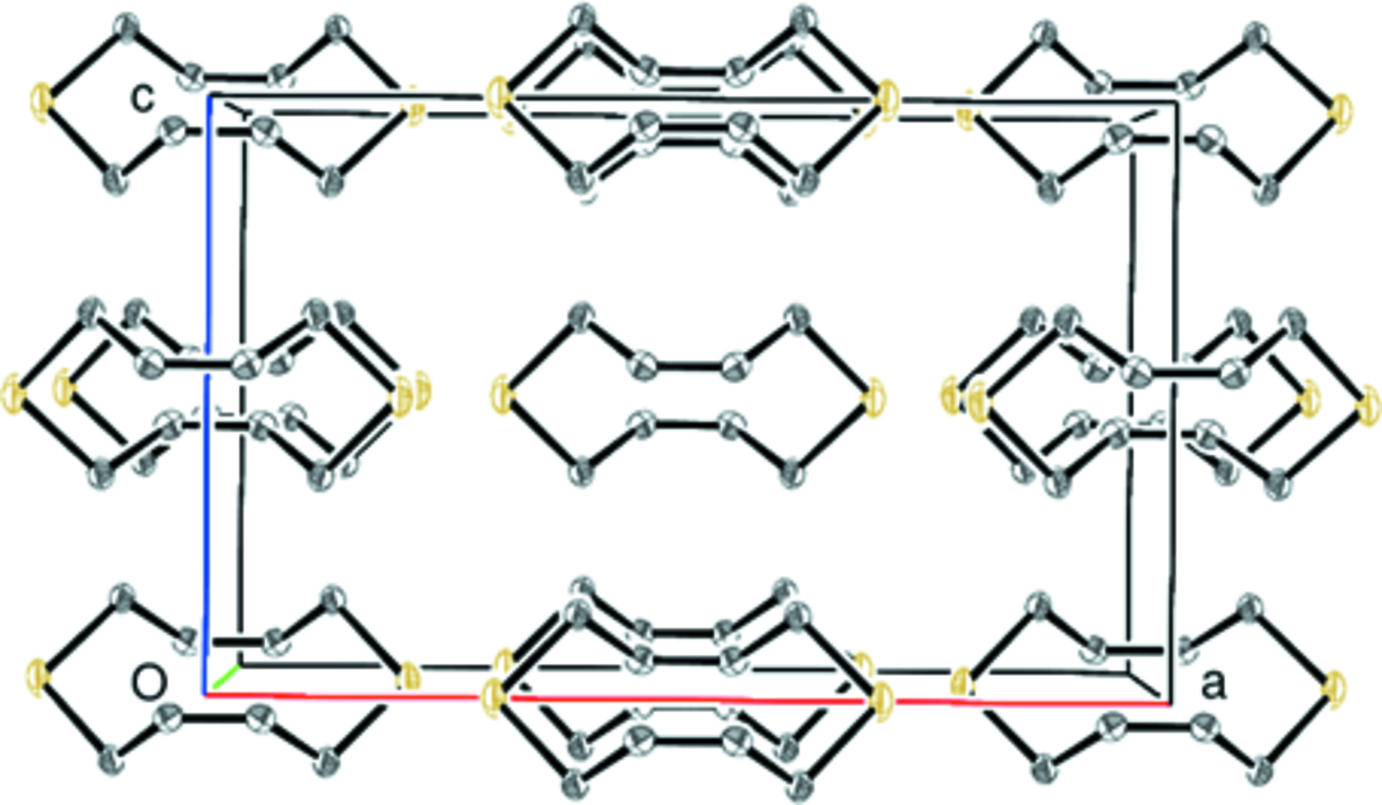

Supplement: Supplementary file 5 [file e-70-o1218-fig2.tif]
